# Effectiveness and Usability of Digital Slow Breathing and Detached Mindfulness Interventions in Late Adolescents: Mixed Methods Study

**DOI:** 10.2196/94578

**Published:** 2026-09-22

**Authors:** Ruya Akdag, Anja van der Voort, Lisa Tessensohn, Julian Versteeg, Simone Vogelaar

**Affiliations:** 1 Department of Cognitive Psychology, Leiden University, Wassenaarseweg 52, Leiden, South Holland, 2333 AK, The Netherlands; 2 Institute of Child and Educational Studies, Leiden University, Leiden, South Holland, The Netherlands

**Keywords:** digital mental health interventions, slow breathing, detached mindfulness, stress, social anxiety, negative metacognitive beliefs, user experience

## Abstract

**Background:**

Many adolescents experience elevated stress and anxiety; yet, access to effective psychological support is limited. Brief digital mental health interventions, such as slow breathing (SB) and detached mindfulness (DM), offer a potentially scalable approach to promoting emotion regulation in daily life.

**Objective:**

This study aimed to investigate whether 1 week of daily practice with a SB or DM intervention reduced stress, anxiety, and negative metacognitive beliefs in youth, and explored participants’ experiences to inform the development of accessible, youth-tailored digital interventions.

**Methods:**

A mixed method approach was used, including quantitative and qualitative data. A total of 147 university students between 16 and 19 years old were randomly allocated to the DM (n=49), SB (n=49), or passive control group (n=49). Participants who were allocated to DM or SB practiced the intervention for 1 week. Stress, anxiety, and negative metacognitive beliefs were assessed at pre- and postintervention using self-report questionnaires. The quantitative data were analyzed with linear mixed-effects models, and we explored daily changes in intervention effect. After posttest, participants were invited to focus groups to evaluate their experience with the interventions (n=37). The qualitative data were analyzed with a content analysis.

**Results:**

Our results showed that a week-long DM intervention significantly reduced stress compared with the control condition. No other differences emerged for the main outcomes on pre- and postmeasurements. However, in the SB group, higher perceived usability was associated with greater reductions in stress. Qualitative findings showed that both interventions were viewed as calming, easy to use, and acceptable, while also highlighting challenges such as maintaining motivation and integrating the exercises into daily routines.

**Conclusions:**

This study provides early formative evidence that brief digital interventions such as SB and DM are feasible and usable, with preliminary indication that DM intervention may support short-term stress reduction. Future research should focus on enhancing motivation and sustained engagement to maximize the effectiveness of digital mental health tools for late adolescents.

## Introduction

### Background

Most people can recall an embarrassing moment in secondary school, perhaps making a mistake in front of their crush or feeling excluded during a group project. These experiences are common, as adolescence is a developmental period characterized by heightened emotional sensitivity and increased stress reactivity [1]. This rise in stress is associated with intensified negative emotional responses and a reduced ability to regulate emotions effectively [2]. As a result, adolescence is considered a critical period for the onset of mental health disorders, particularly generalized anxiety and social anxiety disorder [2]. Social anxiety is one of the most prevalent anxiety disorders among adolescents [3,4] and is characterized by an intense fear of social situations, anticipation of negative evaluation, and fear of humiliation, often leading to avoidance and withdrawal [5,6]. These vulnerabilities are further amplified during periods of societal disruptions, such as economic crises and public health emergencies, which can disrupt social routines, increase uncertainty, and limit access to support [7]. The COVID-19 pandemic is a salient recent example, having significantly increased rates of anxiety and social difficulties among adolescents [8]. As large-scale disruptions have become increasingly prevalent, there is an urgent need for effective and accessible interventions to help adolescents manage stress and anxiety. Importantly, such interventions may be especially valuable early in life, aiming to prevent the progression of subclinical symptoms into more severe anxiety disorders.

Traditional therapeutic approaches, such as cognitive behavioral therapy and exposure-based interventions, have been shown to effectively reduce anxiety [9]. However, these in-person treatments present several barriers to accessibility, including limited awareness of symptoms among adolescents, parents, and teachers, as well as stigma, long waiting lists, high costs, and transportation difficulties [10-18]. As a result, many adolescents who could benefit from support do not access it. Digital mental health interventions (DMHIs) offer a promising solution by providing scalable and easily accessible mental health support. However, most DMHIs currently available on the market lack evidence-based treatments, making them potentially harmful [19].

The modes that fall under DMHI are, for example, internet programs, mobile apps, and virtual reality. DMHIs provide the opportunity to autonomously, independently, and with high accessibility get in touch with interventions that would usually be out of reach [20]. In this study, we focus on 2 specific interventions, that is, slow breathing (SB) and detached mindfulness (DM). They were selected for their strong theoretical and empirical foundation in regulating stress and anxiety [21,22]. Both interventions address key mechanisms implicated in social anxiety, which include maladaptive metacognition and dysregulated autonomic function [23,24], while being well-suited for digital implementation. DM, derived from metacognitive therapy, encourages individuals to observe their thoughts without engaging with them, reducing excessive self-focus and negative metacognitive beliefs [24]. SB directly targets autonomic regulation by increasing heart rate variability (HRV), a key physiological marker of emotional resilience [25,26]. By promoting a breathing pattern with prolonged exhalation, SB enhances parasympathetic activity, fostering relaxation and reducing physiological arousal in stressful situations [27].

Research has demonstrated their effectiveness in both immediate and long-term stress reduction. For example, a recent review of diaphragmatic breathing studies in children and adolescents (6-18 y) found that breathing interventions alone or combined with other therapeutic methods significantly lowered perceived stress, and in many cases also reduced anxiety and depression symptoms over the course of multiweek programs [28]. Moreover, a video-delivered slow diaphragmatic breathing curriculum, designed for high-school students and delivered 3 times per week over 5 weeks, also proved feasible and well-tolerated; students rated the program as useful and easy to follow [29]. Similarly, a systematic review of mindfulness-based interventions for adolescents found consistent improvements in cognitive performance, resilience to stress, and overall well-being, with intervention durations typically ranging from 4 to 16 weeks. Effect sizes for stress reduction ranged from moderate to substantial [30].

Recently, both interventions that we test in this study were evaluated in a healthy sample of 120 young adults using a randomized experimental design, in which participants completed a digitally delivered intervention immediately before performing a public speaking task, an established social stressor [31]. Both DM and SB significantly reduced stress and state social anxiety compared with a control condition. The DM intervention also significantly reduced negative metacognitive beliefs compared with the control condition. Moreover, both interventions were rated as highly acceptable in their digital format, supporting the feasibility of brief, app-based delivery [31]. These characteristics, along with their structured, self-guided nature and standardized delivery protocols, make both interventions particularly suitable for digital implementation. While the previous study established the immediate efficacy of both interventions under controlled laboratory conditions, this study extends their effectiveness and acceptability in adolescents during daily life, using a longitudinal design. This allows us to explore how adolescents engage with the interventions in naturalistic settings, and how they perceive their usefulness, usability, and relevance. These insights are critical for tailoring future versions of the interventions to better fit the specific needs and preferences of this age group.

A major challenge in DMHIs is maintaining user engagement and adherence. High dropout rates and low adherence can significantly undermine the effectiveness of these interventions, limiting their real-world impact [32]. While DMHIs offer accessible and evidence-based mental health support, many struggle to fully engage adolescents, leading to high attrition rates [33]. This disengagement suggests that even if an intervention is effective, its benefits may not be fully realized if users disengage prematurely. Therefore, it is crucial to understand the factors that contribute to attrition and identify strategies to enhance user engagement. Adolescents may disengage due to a lack of motivation, difficulty integrating the intervention into their daily routines, or a perceived lack of immediate benefits. Therefore, in this study, we did not only examine the effectiveness of SB and DM but also explore how adolescents experience and interact with these interventions.

### Objectives of the Study

This mixed methods study aimed to assess the feasibility and preliminary effects of a 1-week digital intervention using SB or DM in reducing stress, social anxiety levels, and maladaptive metacognitive beliefs in late adolescents compared with a passive control. We hypothesized that both interventions led to a significant reduction in social anxiety, stress, and maladaptive metacognitive beliefs from baseline to the 1-week posttreatment measurement compared with the control group. Additionally, we aimed to examine how these intervention effects were moderated by perceived usability and higher motivation invested in the interventions. Furthermore, we explored day-to-day changes in stress, anxiety, and negative thoughts following each intervention session.

Beyond the quantitative assessment, we aimed to gather late adolescents’ perspectives on key aspects of the interventions. Specifically, we explored (1) whether the interventions are feasible and fit into their daily routines, (2) their motivation to engage with the interventions consistently, and (3) their perceptions of the interventions’ enjoyment and clarity. Additionally, we identified which intervention features are most appealing, which aspects are less engaging, and what improvements could enhance their usability and its effectiveness. Furthermore, we explored how to optimize their implementation in a digital format.

## Methods

### Study Design

The study used a mixed method approach [34]. For the quantitative part, a randomized controlled trial was used, with a 2×3 within or between-subject design. Participants went through 2 phases in the experiment (within-subject variable)—the baseline and postintervention phases. Additionally, participants were randomly allocated to one of the following 3 different conditions (between-subjects factor): DM, SB, or the passive control condition. For the qualitative part, participants took part in a focus group in which they could share their thoughts and cocreate new perspectives that might otherwise be hidden [35]. There were 4 focus groups per intervention condition (8 total). Only the 2 intervention conditions took part in the qualitative phase; all 3 were included in the quantitative phase. As our study was also used as input for a study on the experiences with online and live focus groups, both formats were organized (in Microsoft Teams and in the university building), and participants were randomly allocated to one of them.

### Participants

University students were recruited from Leiden University via the online recruitment platform and flyers. Inclusion criteria were: (1) aged 16‐19 years, (2) enrolled as first-year Bachelor students at Leiden University, and (3) proficient Dutch comprehension (C1 level). Exclusion criteria were (1) current or past psychological or neurological disorders, and (2) uncorrected vision problems. A total of 232 participants met the eligibility criteria and provided informed consent (mean age 18.34, SD 1.03 y; n=19, 8.05% male).

Analysis in G*power showed that we needed a sample of 114 participants in total (38 participants per condition; assuming repeated measures, within-between interaction, a small to moderate effect size (*f*=0.18), and a power of 0.80). We intentionally overrecruited to account for expected attrition [36-39].

Participants were randomly allocated to 1 of the 3 conditions (49 per group). Random sequence generation was performed using a computerized procedure before data collection, with participants assigned to a predetermined random order upon sign-up. Allocation was concealed until after baseline questionnaire completion. If they were assigned to one of the intervention conditions, the same email also contained the day 1 intervention link. Due to the nature of the interventions, participant blinding was not possible; however, data analysts were blinded to condition assignment. Those who did not complete the baseline questionnaire were excluded. Both intervention groups practiced their assigned exercise for 7 days. Those who completed at least 6 days were invited to complete the posttreatment questionnaire. Analyses were conducted on a per-protocol basis, resulting in a final analytical sample of 114 participants (retention rate 77.6%). Participants in the intervention groups who completed the postquestionnaire were subsequently invited to a focus group. A total of 37 participants participated across 8 focus groups with group sizes ranging from 3 to 8 participants.

### Ethical Considerations

The study was approved by the Psychology Research Ethics Committee at Leiden University (2024-10-08-R. Akdag-V2-5666). The research procedure complies with the ethical standards of the national and institutional committees and is in accordance with the Declaration of Helsinki. Participants received information about the study’s purpose, procedures, risks, and benefits, and could withdraw at any time. Data were anonymized before analyses, and identifiable information was securely stored, accessible only to authorized personnel. Deidentified datasets were used for analyses to protect privacy. Participants were gifted 6 credits in total if they completed the pre- and postmeasurements, the intervention, and the focus group. The hypotheses, preprocessing of the data, and statistical analyses of the study were preregistered on OSF.

### Procedure

After providing informed consent, participants received a link to the premeasurement questionnaire via email, which they were asked to complete within 1 week using Qualtrics. Upon completion of the premeasurement, participants were informed about the condition to which they had been assigned. Participants in either the DM or SB condition received a daily email for 7 consecutive days containing a link to the online intervention and a brief daily questionnaire. After completing the intervention for at least 6 days, participants received a link to the postmeasurement Qualtrics questionnaire. The pre- and postmeasurements took participants approximately 20 minutes to complete. A suitable date for the focus groups was selected using a date picker tool.

### Intervention

#### DM

**Figure 1.**
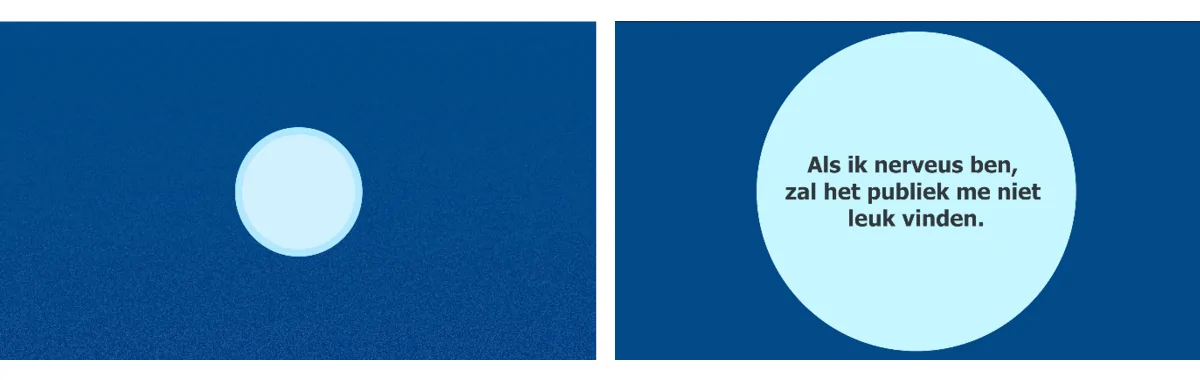
Depiction of the standardization of both the slow breathing (left panel) and detached mindfulness intervention (right panel). The translation of the text is: “When I feel nervous, the public will not like me.”

The DM intervention was based on Wells’ techniques [24]. On the first day, participants watched a video. The first part (2 min) explained the concept of DM using the “busy road” metaphor, illustrating how thoughts can be observed without engagement. The second part (8 min) guided participants through a free-association task, where they were shown common stressful thoughts for their age group but were encouraged to write down their own and use them for the exercise (Figure 1A). During this practice, they learned to observe their thoughts as passing by rather than interacting with them. From the second day onward, the intervention consisted only of the free-association practice, with a reduced duration of 5 minutes per day.

#### SB

The SB intervention began with a 10-minute instructional video on the first day. Participants were introduced to SB techniques through an animated ball that visually guided them through phases: inhalation, breath hold, and exhalation (Figure 1B). They were instructed to breathe diaphragmatically at a rate of 6 breaths per minute, with a prolonged exhalation phase, following established protocols for regulating physiological arousal [25,26]. To accommodate individual preferences, a sound cue was added, allowing participants to close their eyes while following the breathing exercise. From the second day onward, the intervention consisted only of the guided breathing exercise, lasting 5 minutes per day, mirroring the structure of the DM intervention.

#### Standardization of Interventions

Both interventions were designed to be as comparable as possible, ensuring that any observed effects could be attributed to the distinct mechanisms of each practice rather than extraneous differences. The interventions shared the same visual presentation, featuring a dark blue background with a centered light blue ball. Identical background sound cues were used to maintain consistency, and both interventions were administered for the same duration of 5 minutes per day from the second day onward. This duration was chosen based on evidence suggesting it as the minimum effective dose for increasing HRV in SB and reducing negative affect in DM [40,41]. While the SB intervention used the ball’s rhythmic movement to guide breath timing, the DM intervention displayed stressful thoughts inside the ball to encourage nonengagement.

#### Passive Control

Participants in the control condition did not receive any intervention during the 7-day period.

### Primary Outcome Measures

#### Stress Levels

Stress levels were measured during pre- and posttest with the Adolescent Stress Questionnaire – Short [42]. This questionnaire (eg, “I worry about my future”) has 27 items, ranging from 1 “not stressful at all” or “not applicable to me” to 5 “very stressful.” The questionnaire has 9 subscales—home life, school performance, school attendance, romantic relationships, peer pressure, teacher interaction, future uncertainty, school or leisure conflict, and financial pressure. Moreover, 2 extra items were added about health stressors (eg, health of a family member), as these stressors have shown to be relevant for adolescents [43]. The total stress score is derived by computing the total score of the 29 items, with a higher score indicating a higher stress level. The Cronbach α of the premeasurement was 0.87 and of the postmeasurement 0.91.

#### Social Anxiety

Social anxiety was measured during pre- and posttest with the social phobia scale of the Revised Child Anxiety and Depression Scale [44]. Participants have to indicate on 9 items (eg, “I worry when I think I have done poorly at something; Cronbach α_premeasurement_=0.84, Cronbach α_postmeasurement_=0.84), how often the anxiety statements are related to them from 1 “never” till 4 “always.” A higher score indicates a higher social anxiety level.

#### Maladaptive Metacognitive Beliefs

Maladaptive metacognitive beliefs were measured during pre- and posttest with the metacognitions questionnaire – adolescents [45,46]. Participants have to indicate for 6 items on the negative beliefs about worrying subscale how much they agree with statements about their thoughts (eg, “Worrying is bad for me.”; Cronbach α_premeasurement_=0.88, Cronbach α_postmeasurement_=0.88). The scale is rated from 1 (“do not agree”) to 4 (“completely agree”). A higher score indicates a higher negative metacognitive belief.

#### Daily Measurements

Participants in the intervention groups also completed brief daily assessments to capture immediate, session-level responses to the exercise. On day 1, they rated how helpful they thought the intervention would be, which was coded as their outcome expectancy. Each day, they rated their effort during the intervention using 3 adapted items from the Dutch Inzettool (eg, “Today I liked doing the exercise”) [47]. Participants also reported their daily stress level before the exercise and indicated to what extent the intervention reduced their stress, anxiety, and negative metacognitive thoughts afterward. These daily measures were included to explore short-term, within-person patterns of change across the week. A detailed description of all items and scoring procedures can be found in Multimedia Appendix 1.

#### Focus Groups

The topic guides for the focus groups were developed collaboratively by the research team based on the study aims and core domains, including (1) whether the interventions are feasible and fit in their daily life, (2) understanding the level of motivation to practice the interventions daily, (3) determining whether participants find the interventions enjoyable, (4) identifying any additional features or elements that participants believe would enhance the effectiveness of the interventions, and (5) exploring any aspects of the interventions that participants find unnecessary or counterproductive. Draft questions were refined through iterative team discussion to ensure appropriateness for late adolescents. Each section of the guide included open-ended questions with optional prompts to encourage elaboration. The topic guide can be found in Multimedia Appendix 2.

Each focus group was facilitated by 2 trained student researchers, alternating as the moderator or the observer. The moderator opened each session by outlining the procedures and ensuring all participants could contribute, while the observer recorded group dynamics. Each focus group lasted up to 90 minutes and was audio- and video-recorded to support accurate transcription.

### Statistical Analyses

#### Preliminary Analyses

We calculated descriptive statistics (means and SDs) for all study variables. To assess the success of randomization, we compared demographic characteristics (gender, age, and education) and baseline scores for stress, anxiety, and negative metacognitive beliefs across intervention conditions. For categorical variables, chi-square tests were used; for continuous variables, 1-way ANOVAs were conducted. Baseline differences between conditions in perceived usability (System Usability Scale [SUS]) and technology acceptance (technology acceptance model [TAM]) were also examined using 1-way ANOVAs.

#### Quantitative Analyses

We computed 3 separate linear mixed effects models in R (version 4.4.1; R Core Team) to investigate the impact of the interventions on the main outcome variables, that is, anxiety, stress levels, and negative metacognitive beliefs. The models included the within-subject predictor phase (pre- and postdata) and between-subject predictor (ie, condition; control condition was used as the reference category), and their interaction as fixed effects. Level 1 included measurement (ie, pretest and posttest for the 3 of the models). At level 2, participant ID was added as the random intercept. Random slopes were initially included but removed when they were not significant or caused convergence problems with the models. To facilitate effect size interpretation, all continuous variables were standardized before analysis, such that fixed effect estimates (*β*) reflect standardized effect sizes in SD units. In line with the preregistered analysis plan, we applied a Bonferroni correction for the 2 planned comparisons, setting the significance threshold at a corrected α of .025.

#### Exploratory Analyses

To examine day-to-day change across the 7-day intervention, we fit Bayesian multilevel models of stress, anxiety, and negative metacognitive beliefs, with total effort as a predictor of change. We also ran exploratory regressions testing whether usability, technology acceptance, internet use, or digital familiarity predicted pre-post change within each group. Full details and results are in Multimedia Appendices 1 and 3.

#### Qualitative Analyses

We conducted a conventional content analysis to analyze the transcripts of the 8 focus groups [48]. Specifically, we chose this inductive approach because there is limited previous qualitative research on this topic in this age group. Based on the content of the focus groups, codes were generated and organized in categories that gave insight into the experiences of the participants. Moreover, 6 trained research assistants transcribed the focus group data. Each transcript underwent a secondary review for accuracy by a different team member to ensure completeness and fidelity to the original audio. Any discrepancies identified during this review were resolved through discussion, with reference to the original recordings when necessary.

The transcripts were imported into ATLAS.ti (version 25.0.1; Lumivero) and 1 trained research assistant independently performed initial line-by-line coding. Through iterative team discussions involving 2 main researchers and 2 qualitative experts, codes were refined and merged across multiple sessions until consensus was reached. This process produced a final codebook comprising 3 major categories—usability, intervention factors, and improvement suggestions. This process produced a final codebook comprising 3 major categories—usability, intervention factors, and improvement suggestions—each housing multiple codes. Examples of quotations were selected to provide a clear representation of the majority of participants’ opinions. Each quote is labeled by intervention type (ie, DM and SB), followed by the focus group number (1-4), and the individual participant within the focus group.

## Results

### Preliminary Analyses

**Figure 2.**
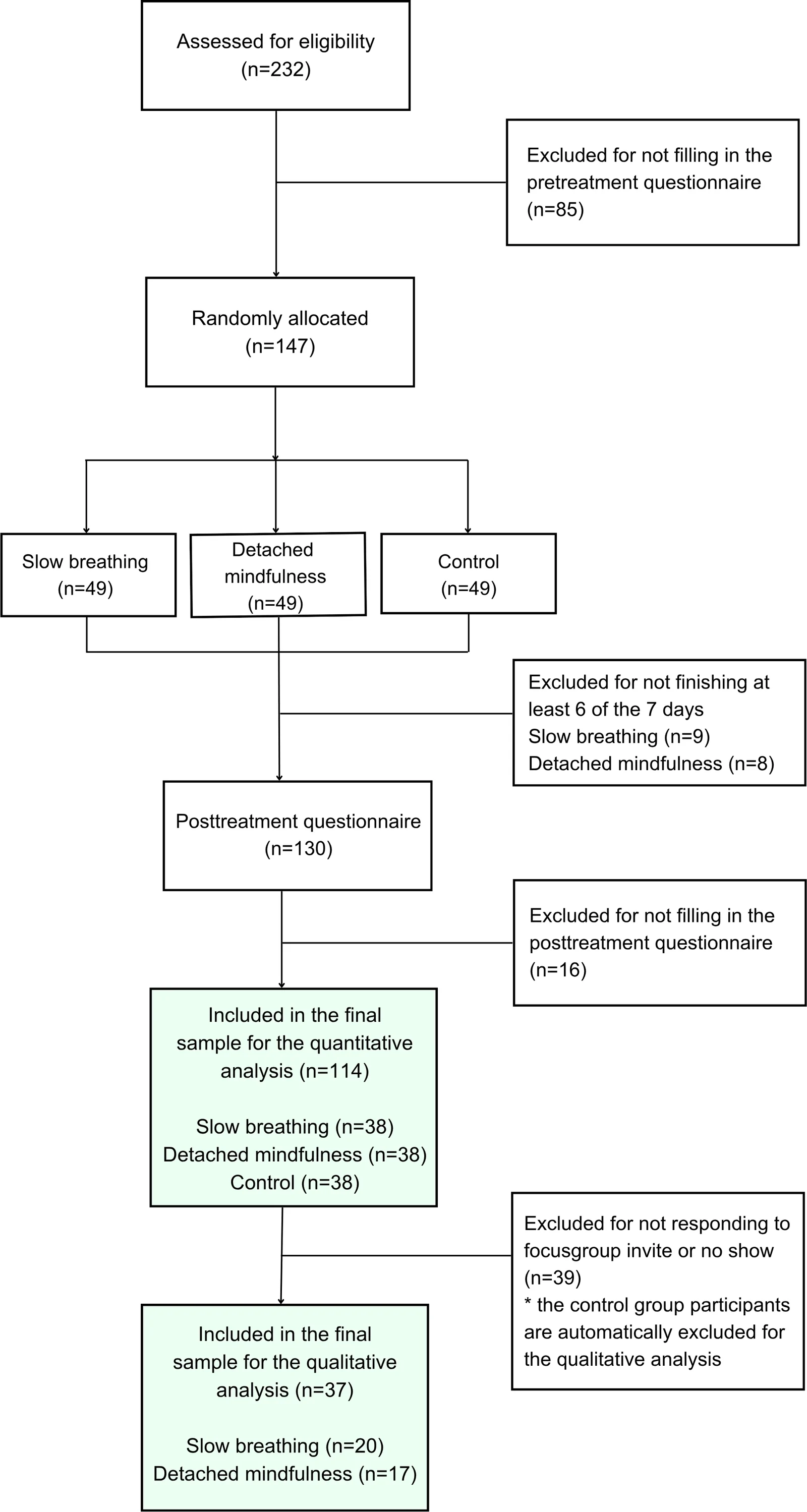
Schematic overview of the flow of the participants through the study procedure including the final sample size for our quantitative and qualitative analyses*.*

A total of 232 potential participants were assessed for eligibility, with 147 meeting the criteria and being enrolled in the study. A detailed overview of participant enrollment is illustrated in Figure 2. To assess whether randomization was successful, we compared demographic variables and trait measures across conditions. There were no significant differences between conditions in gender, age, and education level (all *P* values>.338). No differences were observed in baseline levels of anxiety and negative metacognitive beliefs across all conditions (all *P* values>.134). However, we did find a significant difference between the conditions on stress scores (*P*<.001). Specifically, the participants in the DM condition scored higher on stress than both the control and SB conditions. On average, participants scored 59.98 on overall stress, which is below the midpoint of the scale (81) and suggests relatively low to moderate levels of self-reported stress in this nonclinical sample. Additionally, participants scored average (mean 22.38, SD 4.81) on anxiety levels and negative metacognitive beliefs (mean 14.09, SD 4.09).

Participants in both intervention groups rated the intervention as highly usable (mean_SB_=36.63, SD 4.70 and mean_DM_=33.2, SD 6.12 on the SUS) and moderately acceptable (mean_SB_=17.3, SD 4.59 and mean_DM_=16.67, SD 4.04 on the TAM). On average, participants in the SB group gave the intervention a grade of 7.1 out of 10 and the participants in the DM group a 6.5. The majority (n=67, 89.2%) provided a positive evaluation, describing the intervention as calming, helpful in reducing stress, and easy to follow. A smaller subset (n=9, 10.8%) expressed negative views, noting that the intervention felt repetitive, was difficult to concentrate on, or was not personally effective. A full overview of descriptive statistics is provided in Multimedia Appendix 4.

### Feasibility Outcomes

Regarding recruitment, of the 232 individuals assessed for eligibility, 147 completed the pretest, a recruitment rate of 63.4%. Although this falls below the benchmark of 70% [49], it is consistent with a systematic review and network meta-analysis indicating that attrition is a common challenge in internet-based interventions [50]. Of the 147 who completed the pretest, 114 also completed the postmeasurement, resulting in a retention rate of 77.6% that exceeds the 70%-76% benchmark [49]. Adherence to the daily exercises was high in both intervention conditions. In the SB group, participants completed on average 6.3 of the 7 days, with 88% (n=33) completing at least 6 days. In the DM group, participants completed on average 6.0 days, with 78% (n=29) completing at least 6 days. Finally, no technical problems were reported during any intervention, a technical issue rate of 0%.

### Quantitative Analyses

#### Pre-Post Intervention Effect on Stress

**Figure 3.**
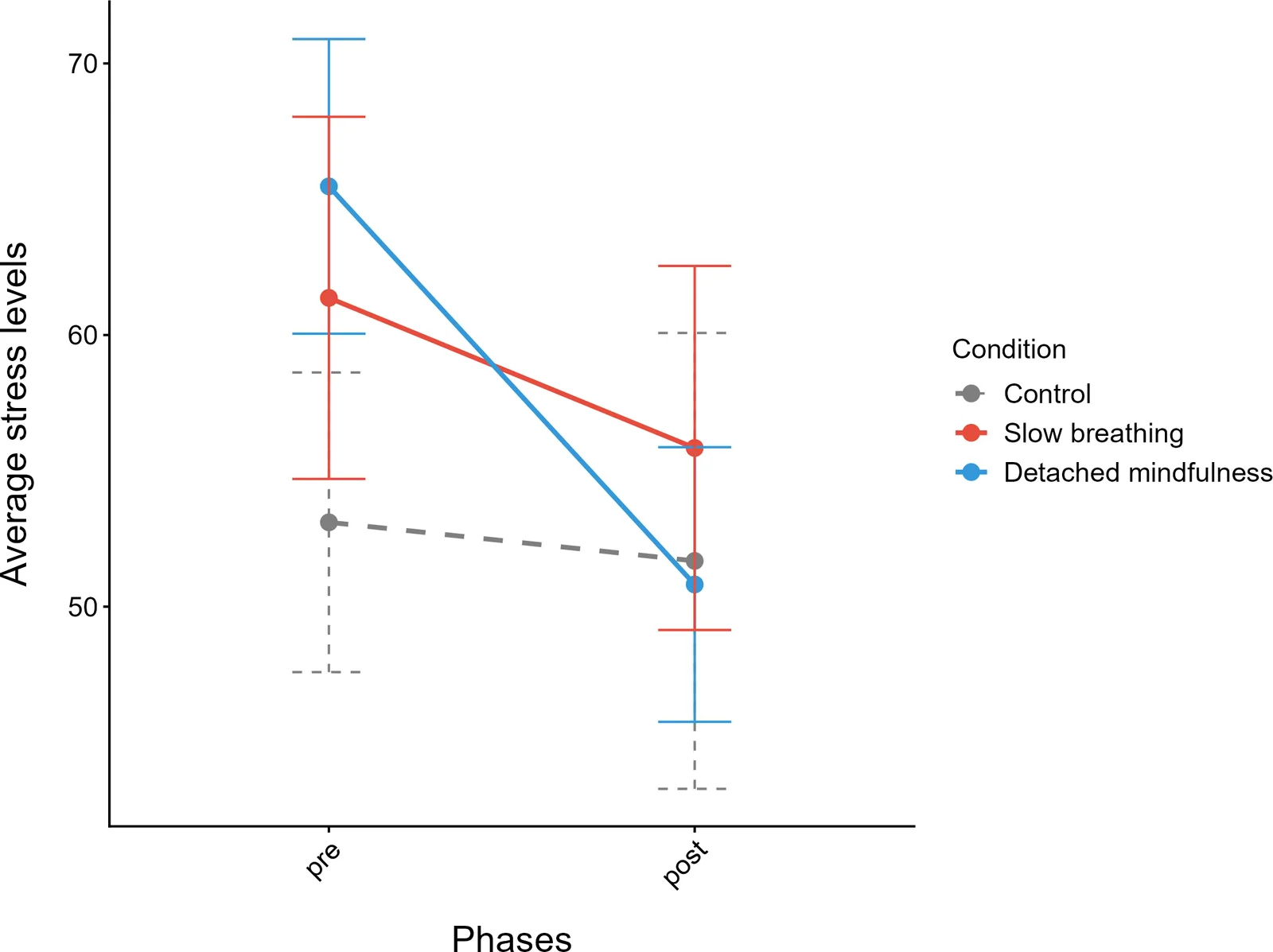
Stress from preintervention to postintervention for different conditions (N=114).

A linear mixed-effects model was used to assess the effects of condition (control, SB, and DM), time (pre vs post), and their interaction on stress scores. We found a significant main effect of time, *F*_1, 228_=17.424, *P*<.001 and, as expected, a significant interaction effect between condition and time *F*_2, 228_=5.141, *P*=.007. Participants showed lower levels of stress in the posttest measure compared with the pretest measure and this decrease was significantly larger for the participants in the DM condition compared with the control group. No significant main effect of condition was found (*P*=.09). Refer to Table 1 for the full model output and Figure 3 for a progression overview.

To examine whether the stress reduction observed in the DM condition could be attributed to regression to the mean, we conducted a sensitivity analysis using ANCOVA. As this sensitivity analysis examined a single comparison (DM vs control), no correction for multiple testing was applied and a conventional α level of .05 was used. Postintervention stress scores were regressed on condition and baseline stress scores. After controlling for baseline stress, the DM condition continued to show significantly lower postintervention stress compared with the control condition (*β*=−8.73, SE=4.31; 1-tailed *t*_228_=−2.02; *η*²=0.01; *P*=.05). The baseline stress score was a significant predictor of postintervention stress (*β*=.64, SE=0.09; 1-tailed *t*_228_=6.85; *η*²=0.30; *P*<.001).

**Table 1.** Results of the linear model predicting stress by condition, time, and their interaction (N=114). The control condition and preintervention time point are the reference categories.

Predictor	*β* (SE)	95% CI	*t* test (*df*)	*P* value
Condition category				
Slow breathing	.073 (0.146)	−0.214 to 0.360	0.500 (228)	.62
Detached mindfulness	.109 (0.148)	−0.180 to 0.399	0.740 (228)	.46
Time				
Post	−.069 (0.145)	−0.353 to 0.215	−0.476 (228)	.64
Interactions				
Slow breathing×post	−.199 (0.205)	−0.602 to 0.203	−0.971 (228)	.33
Detached mindfulness×post	−.643 (0.205)	−1.045 to −0.241	−3.132 (228)	.002^a^

a *P*<.01.

#### Pre-Post Intervention Effect on Anxiety

**Figure 4.**
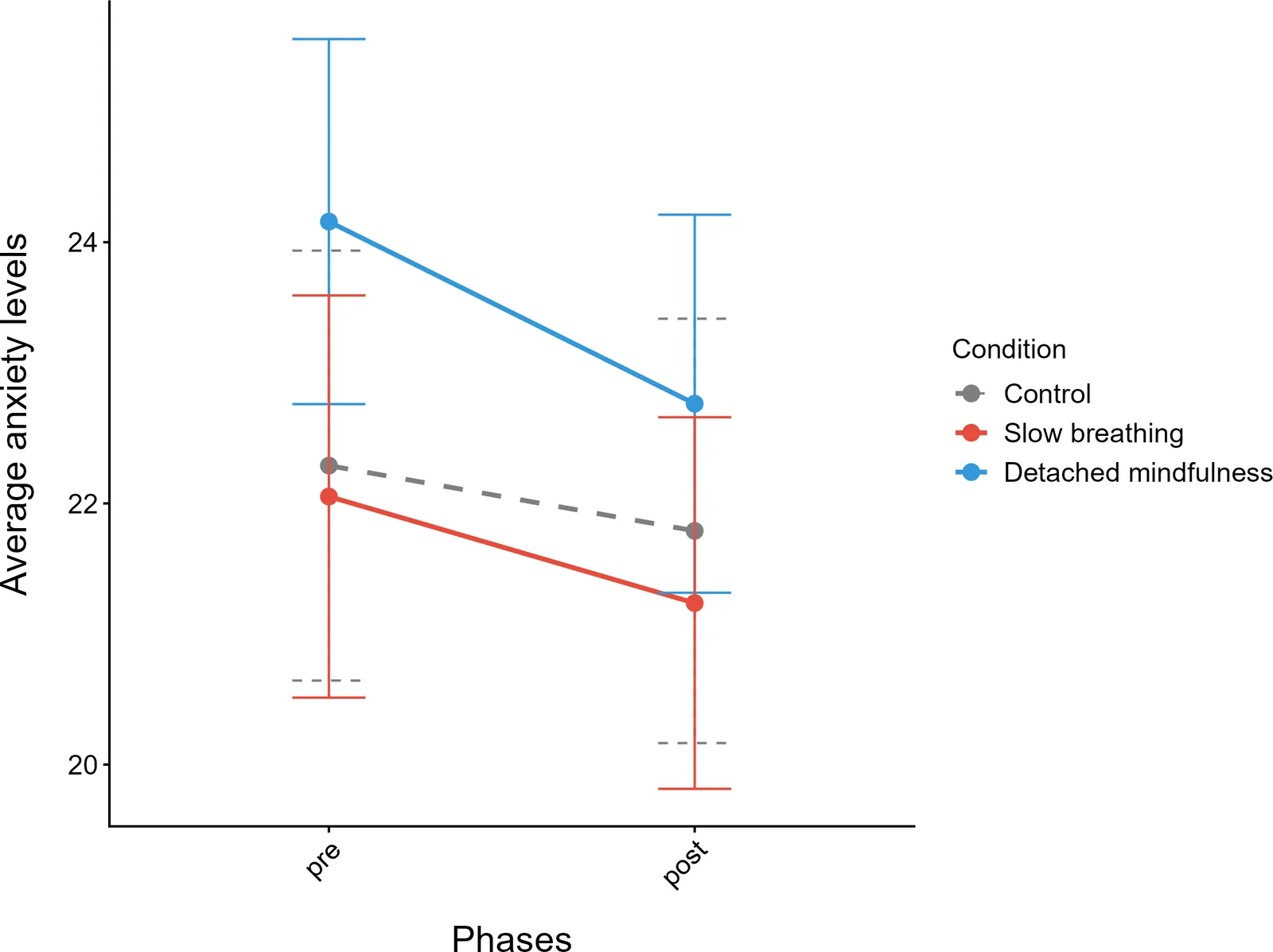
Anxiety from preintervention to postintervention for different conditions (N=114).

A linear mixed-effects model was used to assess the effects of condition (control, SM, and DM), time (pre vs post), and their interaction on general anxiety symptoms. The model revealed no significant main effects of condition, time or the interactions (Table 2 and Figure 4). This finding suggests that none of the intervention groups showed a differential change in anxiety scores from pre- to posttest compared with the control group.

**Table 2.** Results of the linear model predicting anxiety by condition, time, and their interaction (N=114) Note. The control condition and pre-intervention time point are the reference categories.

Predictor	*β* (SE)	95% CI	*t* test (*df*)	*P* value
Condition category				
Slow breathing	−0.049 (0.225)	−0.490 to 0.391	−0.219 (146)	.83
Detached mindfulness	0.389 (0.225)	−0.052 to 0.829	1.731 (146)	.09
Time				
Post	−0.104 (0.113)	−0.325 to 0.117	−0.921 (114)	.36
Interactions				
Slow breathing×post	−0.066 (0.160)	−0.379 to 0.247	−0.411 (114)	.68
Detached mindfulness×post	−0.186 (0.160)	−0.499 to 0.127	−1.166 (114)	.25

#### Pre-Post Intervention Effect on Negative Metacognitive Beliefs

**Figure 5.**
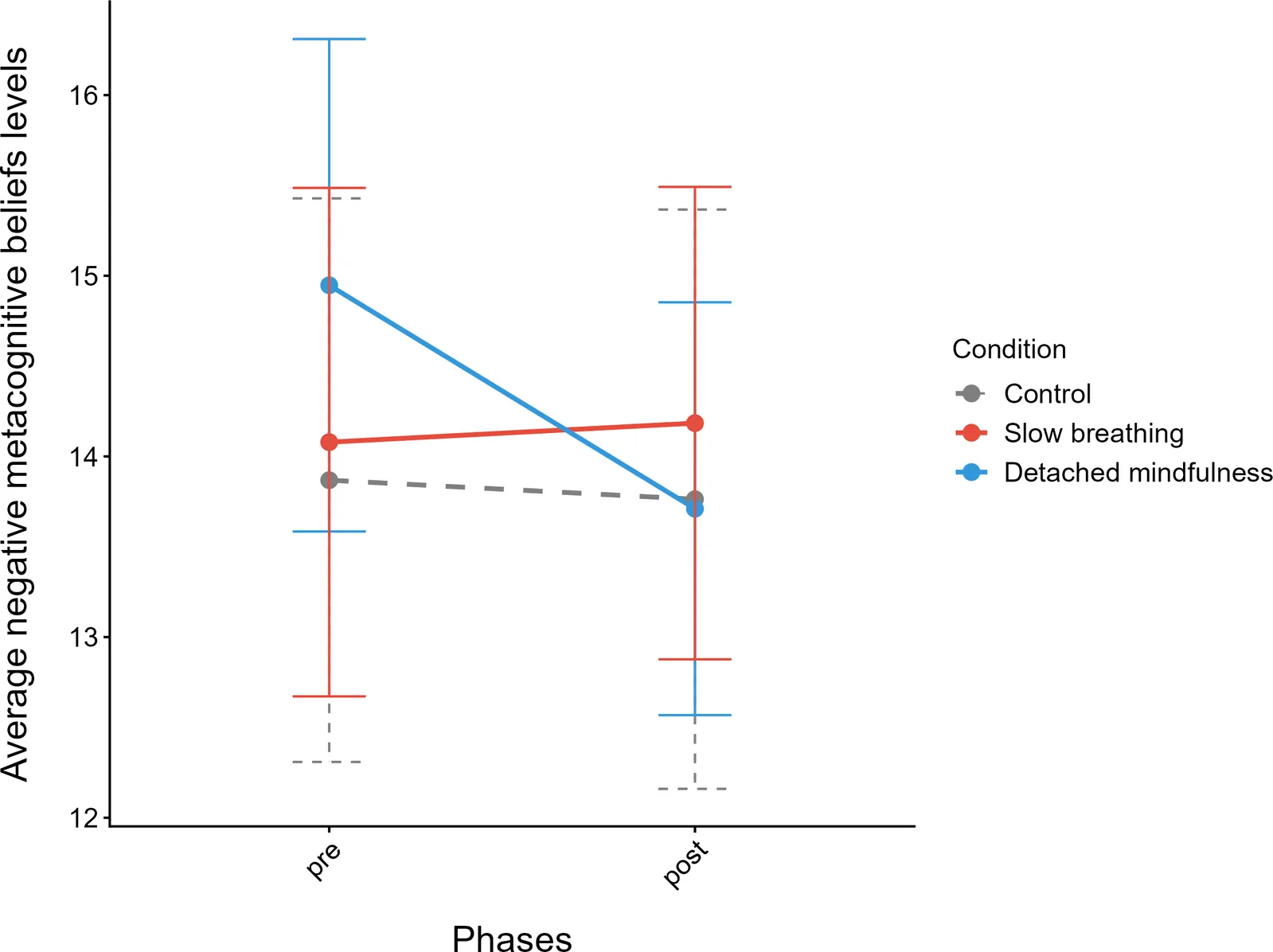
Negative metacognitive beliefs from preintervention to postintervention for different conditions (N=114).

A linear mixed-effects model was conducted to examine the effects of condition (control, SM, and DM), time (pre vs post), and their interaction on negative metacognitive beliefs. The model revealed no significant main effect of time and condition (Table 3 and Figure 5).

**Table 3.** Results of the linear model predicting negative metacognitive beliefs by condition, time, and their interaction (N=114). Note. The detached mindfulness condition and pre-intervention timepoint are the reference categories.

Predictor	*β* (SE)	95% CI	t test (*df*)	*P* value
Condition category				
Slow breathing	0.048 (0.228)	−0.399 to 0.495	0.210 (137)	.83
Detached mindfulness	0.246 (0.228)	−0.201 to 0.692	1.078 (137)	.28
Time				
Post	−0.024 (0.099)	−0.217 to 0.169	−0.243 (114)	.81
Interactions				
Slow breathing×post	0.048 (0.140)	−0.226 to 0.321	0.343 (114)	.73
Detached mindfulness×post	−0.258 (0.140)	−0.531 to 0.016	−1.846 (114)	.07

### Qualitative Results

#### Overview

To complement the quantitative results, we conducted a qualitative analysis of postintervention focus groups to better understand late adolescents’ experiences. In the analysis, we focused on participants’ experiences with the intervention and their motivation. In total, three overarching categories were identified: (1) usability and engagement, (2) perceived intervention effects, and (3) suggestions for improvement. For the first 2 categories, points of evaluation as well as points of improvement were coded for existing parts of the interventions. The third category consisted of improvement suggestions for add-ons that were proposed by us. An overview of categories and subcategories is provided in Table 4 and Table 5.

**Table 4.** Summary of categories across both conditions and participants’ solutions.

Main category and subcategories	Detached mindfulness (DM)		Slow breathing (SB)	
	Evaluation	Improvements	Evaluation	Improvements
Usability				
Ease of use	Easily accessible, straightforward, and minimalist design, difficult instructions	Could benefit from more concrete instructions	Easily accessible, straightforward, and minimalist design	—^a^
Focus and engagement	Diminishes with perceived lack of effectiveness, forceful daily obligation, lack of variation in exercise	Variability in exercises	Diminishes with perceived lack of effectiveness, forceful daily obligation, lack of variation in exercise	Variability in exercises
Structural aspects	Layout and reminders very positive	—	Layout and reminders very positive, auditory aspects lack fine-tuning	Customizable sound options, choice whether to enable or disable sound
Intervention effects				
Immediate calming and relaxation	Sense of calmness, nonintrusive nature of the exercise	—	Sense of calmness (especially end of the day), nonintrusive nature of the exercise	—
Perceived effectiveness for stress regulation	Reduced tension when the exercise was understood	Clearer instructions to reduce confusion	Reduced tension and stress, applied during other moments in the day	—

a Not applicable.

**Table 5.** Summary of added feature suggestions for both conditions

Main category and subcategories	Evaluation
Suggestions for added features	
Leaderboard	Competitiveness negatively perceived as more stress inducing, mental health seen as something personal and private
(AI) Chat Feature	AI Chat not interesting, unless either replaced by human health professional, or reinforced by human health professional by means of forwarding questions from AI to human
Progression	Generally positive, but loss of streaks could lead to negative feelings and loss of motivation
Peer Function	Minimal interest to share experience with other users, emphasis of personal aspect regarding mental health. However, not feeling like the only one is appreciated (collective experience)

#### Category 1: Usability

##### Ease of Use

Participants in both intervention conditions reported being satisfied with the ease of use of the intervention. Across focus groups, participants mentioned that the intervention was easily accessible, with minimal difficulties navigating the intervention exercise and completing the tasks, highlighting the straightforward design. As 1 participant in the SB condition summarized:

*Yes, I found it very easy actually. It was very easily accessible, and clear as to what the task was. Very accessible, via multiple devices.*

[SB2-2]

Similar sentiments were echoed about the exercise in the DM condition. Participants appreciated the minimalist design of the intervention which made focusing more easy:

*Yes it was very basic, so it was not all that distracting, which helped.*

[DM5-2]

*It was fairly simple. So, nothing to criticize.*

[DM5-1]

While overall usability was rated positively, some participants in the DM group noted challenges with the clarity of the instructions during the exercise itself. These difficulties were not related to the navigation to the intervention exercise, but rather to the lack of concrete instruction on how to correctly perform the mindfulness task. Specifically, participants expressed difficulty with understanding the instruction “to step back from their thoughts,” a core element of DM that relies on metacognitive awareness and attentional control. For instance:

*Yes, I was talking about, like, the instructions, that you had to experience a particular thought. But that you kind of had to let it go past. I personally found that a bit difficult … I was not really sure whether it was the correct thing*.

[DM1-1]

Another participant elaborated:

*I thought it was nice when they said to think of something else, something positive ... But yeah, I found it difficult to picture myself outside of it ... you will always think of something again.*

[DM4-4]

Relatedly, participants suggested that the guidance was sometimes too abstract, making it harder to apply the technique effectively. As 1 participant explained:

*I think I would have preferred if it was a bit less abstract. From what I can remember, it was just those cars that pass that you then had to look at, but not get into. It would have been better if it would have been a little more concrete with real thoughts and examples.*

[DM1-4]

In sum, while both interventions were viewed as accessible and user-friendly in terms of navigation and interface, some participants, particularly in the DM condition, expressed uncertainty regarding how to follow the abstract cognitive instructions of the task. This feedback suggests that enhancing the concreteness of instructions and offering relatable examples may improve engagement and effectiveness, particularly for late adolescents unfamiliar with DM.

##### Focus and Engagement

While many participants reported no particular difficulty focusing during the exercises, others described challenges in maintaining concentration over time. These difficulties were reported across both the SB and DM conditions. One key factor influencing motivation was the perceived effectiveness of the intervention. Focus and engagement diminishes with perceived lack of effectiveness. As 1 participant expressed:

*I would also not do it [continue the intervention], but that is mainly because I did not experience that the intervention affected me. So, I think I would not be motivated to continue to do that.*

[DM4-1]

Another factor affecting motivation was the sense of obligation tied to daily participation. Some participants felt that the intervention became a chore, particularly when trying to fit it into busy routines:

*I found it more difficult, just because I am not home very often, so to then take a moment to sit, feet on the ground, yes. I found that once I was home, I would rather lay down on my bed and rest*.

[SB2-6]

However, the most frequently cited barrier to both motivation and concentration was the lack of novelty and variation in the intervention over the course of the week. As repetition increased, engagement decreased. One participant noted:

*I found the repetition, the way it works with me is that I thought; Why am I doing this actually? Because while you may get more focused, I would get less focused with each day. Perhaps that the music could have alternated between days… that it would at least make you pay attention again*.

[DM4-1]

Participants consistently noted that the intervention became increasingly predictable because the format, content, and structure were identical each day:

*Well, maybe the fact that every day was the same. That I thought at a certain point, I’ve seen it all. I’d prefer some more variation in the [instruction] videos.*

[DM7-3]

Despite this, some participants appreciated the routine and reported improved experience through repetition:

*You can do it! Don’t think about anything. But that does not work that well of course. It did get better after a few minutes, but it was also the more often I did the exercise, the better it would get*.

[SB6-1]

To address these engagement challenges, many participants suggested increasing variability in future versions of the app. A customizable interface offering a range of SB or DM exercises was seen as a possible solution:

*I think that a bit of variation might be good too, because everyone interacts with these things in a different way... So perhaps something like having different versions will work better for people having difficulties.*

[SB2-7]

Regardless of these motivational challenges, overall perceptions of the interventions remained positive. Participants appreciated their simplicity and ease of use, suggesting that the core usability was not a barrier. However, to enhance long-term engagement, future iterations of these interventions may benefit from introducing variability, such as alternating audio tracks, changing visuals, or incorporating adaptive elements tailored to users’ needs.

##### Structural Aspects

Participants shared a range of perspectives on the structural features of the intervention application, including layout, reminders, and auditory elements. While some components were praised, others revealed clear opportunities for improvement.

The layout, consisting of visuals, font, and color use, was generally well-received. Participants described it as clean and functional, with very few criticisms. One feature that garnered consistent appreciation was the daily reminder system. These notifications helped participants integrate the intervention into their routines:

*I would get an email every day as a reminder that popped up and I would think, ah yes, I will do it right away. Otherwise, I would have probably forgotten, if I am being honest.*

[SB2-1]

Participants explained that reminders were especially helpful on busy days when the intervention might otherwise be forgotten:

*Yeah, I also had a day like that, but it was probably because I had such a busy day, and it’s not really part of your routine yet or anything. So, I kept forgetting it then.*

[SB2-2]

Feedback on the auditory elements, specifically the instructional voice and background music, was more mixed. Although the voice was often described as calming, participants in both conditions reported that it sometimes broke their focus or interrupted the calming atmosphere. In the SB condition, the timing of the voice was a common concern:

*The first few minutes were alright, I could retain the rhythm and keep my head empty. But definitely towards the end it became more and more difficult. Then, the voice recording would say that it is just one minute left, and then I am completely out of it again*.

[SB6-6]

In the DM condition, participants noted that too many overlapping spoken instructions could lead to renewed stress:

*I really felt that, at a certain point she [the voice] would interject and would repeatedly say to try and focus on it [the statement], when I had just done that… and then I thought to myself that another statement just appeared, and I would feel stress again*.

[DM4-3]

Similarly, reactions to the background music varied. While some participants found it relaxing, others experienced it as distracting or even anxiety-inducing:

*I was distracted by it [the music]... Some people might find it relaxing, but for me personally it makes me nervous.*

[DM4-1]

Technical issues with auditory aspects were also noted, particularly the low volume of auditory cues:

*I thought the background sound was annoying. It was said that ‘if you cannot look at the screen, listen to the sound.’ The sound was so quiet that you could barely hear anything.*

[SB2-8]

To improve the auditory and structural aspects of the intervention, participants repeatedly suggested greater customization. Preferred options included toggling background music and instructional voice on or off, adjusting exercise duration, and choosing from different layouts or exercise formats:

*Or something like multiple options between exercises, where some are recommended with instructional voice recordings, and some are recommended without, so you can decide for yourself whichever suits you better… Or that you can turn the music on or off, something like that*.

[SB6-7]

*If you would have multiple options regarding the music or voice, or in terms of exercise duration, maybe a different layout for the dot on the screen, then you can see for yourself where your needs lie, on that day. It’s different for everyone*.

[SB6-2]

In sum, while the app’s structural design was generally sound, participants emphasized the need for flexible, user-driven features to accommodate diverse preferences and improve engagement. Customizability stood out as a core recommendation for future iterations of the intervention.

### Category 2: Perceived Intervention Effects

#### Immediate Calming and Relaxation

Participants across both intervention groups frequently reported experiencing an immediate sense of calmness while engaging with the exercises. This calming effect was one of the most described outcomes and was primarily experienced during or immediately after the intervention. Participants often described the exercises as relaxing, soothing, and pleasant, particularly when used in the evening or after a busy day.

In the DM exercise, calmness was often attributed to the nonintrusive nature of the exercise. Participants noted that the quiet visuals, slow pacing, and background music created a peaceful atmosphere, even when they found the cognitive component of the exercise challenging. As 1 participant noted:

*I did feel a bit calmer, because I just found the video itself very calming … It was nice just to sit still for five minutes and let your thoughts pass by. You didn’t have to do anything else. That made it feel quite peaceful*.

[DM1-4]

Similarly, another explained:

*When I could really focus, I felt like I could put myself aside for a moment. And even when I couldn’t, just listening to the music helped me feel more at ease.*

[DM5-4]

Participants in the SB condition similarly described the intervention as calming, often highlighting bodily sensations such as relaxation and sleepiness:

*It worked pretty well for me. I actually got a bit sleepy, in a good way*.

[SB6-4]

Many explicitly mentioned using the exercise at the end of the day to unwind:

*I found it really nice to just sit quietly and focus on my breathing. I did it before going to sleep and noticed it really helped me feel more relaxed.*

[SB3-1]

*Taking that moment before bed to slow down helped me clear my mind and feel more calm.*

[SB3-4]

Together, these findings indicate that both interventions provided participants with a momentary state of calmness, either cognitively (through detached observation of thoughts) or physiologically (through regulated breathing). This calmness appeared largely situational, linked to the structure and sensory qualities of the exercise rather than to deliberate stress regulation.

#### Perceived Effectiveness for Stress Regulation

In contrast to the consistently reported calming effects, participants expressed more mixed experiences regarding whether the interventions helped them regulate stress. While several participants reported feeling less stressed during or after the exercise, others found the interventions ineffective or, in some cases, stress-inducing. Several participants in the DM group described reductions in stress following the exercise, particularly once it was completed:

*I noticed that once the video was over, I could really leave my stress behind in the video. That’s when I felt the stress had gone down.*

[DM4-3]

However, this was not universal. Some participants experienced the exercise as cognitively demanding, reporting increased stress or confusion during the task itself:

*During the video, I actually got more stressed. I kept being told to focus on my stress, then to let it go, and that made me more panicked. But afterwards, I did feel calmer.*

[DM4-3]

Others felt the exercise was unhelpful when they were already preoccupied or not stressed to begin with, noting that being guided to focus on stressors could worsen their mood:

*For me, it didn’t really do anything. I didn’t experience any stress reduction … In the morning, when I had a lot on my mind, it didn’t help. It actually made me stress more about everything I still had to do*.

[DM4-1]

Some participants felt the intervention was not always relevant, especially on days when they were not stressed or found the guided thoughts too rigid:

*If I wasn’t stressed that day, it didn’t feel very useful.*

[DM5-4]

*It shouldn’t push people to think about something they weren’t already worried about… it can make it worse.*

[DM5-2]

Participants in the SB group more consistently reported perceived stress reduction, particularly in high-stress moments. Some participants reported transferring the technique to daily life, using the exercise before stressful events or during moments of heightened tension, such as presentations or panic-like situations:

*I noticed it really lowered my stress. I did it before a presentation, and just a few deep breaths helped.*

[SB3-1]

*I didn’t feel like doing it at first because I was so stressed, but the fact that it was seven minutes helped me get into it. By the end, I had less stress than when I started.*

[SB8-2]

*It didn’t reduce stress directly, but I did use it when I noticed my breathing getting fast in a panic moment, it helped me calm down.*

[SB8-1]

Still, a few participants noted that the effect was short-lived or only present during high-stress moments:

*It helped me in the moment, but over a whole day I didn’t really feel more relaxed. Maybe you need to do it longer than a week to notice that.*

[SB7-1]

Taken together, these findings suggest a clear distinction between feeling calm during the intervention and effectively regulating stress beyond the exercise. While both interventions reliably induced momentary calmness, they were not consistently experienced as tools for managing stress in daily life. This highlights the importance of clearer guidance on applying the techniques to real-life stressors and supporting late adolescents in translating relaxation during the exercise into meaningful stress regulation.

### Category 3: Improvement Suggestions for Added Features

#### Overview

In addition to the improvement suggestions given by the participants regarding the existing features, participants were also asked how they would feel about the addition of specific features that were not yet present in the digital intervention. In both conditions, participants were asked about 4 possible additions.

#### Leaderboard

First, a public leaderboard that would track the scores of the users. The more exercises completed, the more the score would go up. This way, a feeling of competitiveness would be evoked, possibly allowing for more motivation to keep doing mental health exercises. This was unequivocally detested, in both groups. Many would use a similar reasoning, stating that mental health is something quite private, and not something that should garner competitiveness or comparison to others, as it could even lead to more stress and anxiety. As stated by DM5-1:

*I would find it very unpleasant if I had to compare myself to others, I would get very anxious. I prefer to keep things like this to myself, so no I would not like such a feature.*

#### AI Chat Feature

Second, any form of a chat feature or AI assistant tool. Participants proposed the inclusion of a chat feature within the app that could provide on-demand support for questions related to mental health. While most participants expressed a preference for speaking with a real person rather than an AI-based chatbot, many agreed that such a feature could be valuable if it were operated by a certified mental health professional. As 1 participant explained:

*I don’t know, I would rather talk about my feelings to a real person as opposed to AI.*

[DM7-2]

However, some participants noted that the chatbot would not need to be fully human-operated at all times. They indicated it would be acceptable if the chatbot could automatically escalate more complex or personal queries to a real professional when necessary.

*The only thing is that I would like there to be a real person, that there would be the possibility of the AI forwarding questions to them when necessary.*

[SB8-1]

#### Progress Tracking

The third proposed feature focused on providing users with a way to track their progress over time. Suggestions included implementing daily streaks for completing exercises on consecutive days, a timeline showing how long users had engaged with the app, and visual summaries of changes in stress and anxiety levels. Overall, participants responded positively to the idea of progress tracking, noting that it could increase motivation and reinforce a sense of commitment. However, some also raised concerns about potential downsides. In particular, the use of streaks was seen as potentially counterproductive if missing a day led to frustration or reduced engagement. As 1 participant explained:

*Those daily goals sound positive in and of themselves, but it shouldn’t become a daily streak like you see on Snapchat or Duolingo. Then it starts to feel mandatory. And if you lose a streak after three weeks because you were too busy one day, it just leads to disappointment for failing and having to start all over again.*

[SB2-3]

#### Peer Interaction and Community Features

A final suggestion involved adding a peer interaction or community tab, allowing users to connect with others either during or after completing an exercise. This feature aimed to provide a sense of social support and shared experience in managing mental health. Responses to this idea were mixed. While many participants preferred to keep the intervention private and personal, some saw value in the idea of shared participation. For example, 1 participant emphasized:

*Well, I think this is supposed to be more of a moment to yourself. It really is something you take time out of your day for, for yourself. You decide to do the exercise, and that is your own process, your own progression. This is something you keep to yourself; it would not be something I would share with anyone else.*

[SB6-2]

In contrast, others appreciated the idea of feeling part of a collective experience. One participant noted:

*Maybe you’ve had a really tough day, and then just knowing that other people are also doing these kinds of exercises can actually help you a bit.*

[DM8-2]

*Yes, same here. I think it would be nice to be able to chat about it with others, either during or after, just to share how it went and how you’re experiencing it.*

[DM8-1]

Overall, participants expressed a clear preference for added features that support motivation and reflection without increasing pressure, comparison, or social exposure. Features perceived as competitive or public, such as leaderboards, were consistently rejected due to concerns about privacy and increased stress. In contrast, progress tracking and optional support features were viewed more positively when they allowed for flexibility and personal control. Responses to social and AI-based features highlighted the importance of autonomy, safety, and the option to engage or disengage based on individual needs. Together, these findings underscore that late adolescents value DMHIs that remain supportive, nonjudgmental, and aligned with the personal nature of mental health care.

## Discussion

### Principal Findings

The aim of this study was to investigate feasibility and preliminary effects of brief digital SB and DM interventions on stress, anxiety, and negative metacognitive beliefs in youth. In addition, we explored participants’ subjective evaluations of the interventions to inform future design decisions tailored to this target population. Our quantitative results showed that a week-long DM intervention significantly reduced stress compared with the control condition. No other between-group differences emerged for the main outcomes on pre- and postmeasurements. However, in the SB group, higher perceived usability was associated with greater reductions in stress postintervention. Although there were no clear improvements across the 7-day intervention period overall, day-level analyses revealed that participants who exerted more effort experienced greater benefits from the intervention.

### Intervention Effects and User Experience: Integrated Findings

As hypothesized, participants in the DM condition experienced a significant reduction in self-reported stress compared with the control group after 1 week. The stress-reducing effect of the DM condition aligns with previous work showing that brief metacognitive strategies can reduce emotional reactivity in stressful situations [41]. In the earlier laboratory-based study with young adults [31], the same intervention reduced stress and social anxiety immediately following a social-evaluative task. This study extends those findings to a real-world adolescent sample, suggesting that even brief, self-guided digital training in DM can lead to noticeable reductions in perceived stress across a week.

The qualitative findings help contextualize and strengthen this result. Late adolescents in both intervention conditions frequently described experiencing a sense of calm or temporary relief from stress following the exercises. These brief, guided moments of stillness and sensory focus appeared to offer a pause in participants’ busy daily lives, supporting the idea that even low-intensity digital interventions may offer a subjective sense of relief when actively engaged with. This convergence between quantitative stress reduction and qualitative reports of calm suggests that the interventions were experienced as emotionally beneficial, even when broader symptom change was limited.

It should be noted that the baseline stress levels were relatively low to moderate, raising questions about the practical significance of the observed stress reduction. The findings are therefore best interpreted as proof-of-concept evidence that the intervention can produce measurable stress reduction under naturalistic conditions, rather than as evidence of clinically meaningful change. Future research should examine the intervention in samples with elevated baseline stress levels to better assess the potential practical impact of the mindfulness condition.

Despite the effects on stress, no intervention effects were observed for anxiety or negative metacognitive beliefs compared with the passive control group. One explanation may lie in the short intervention duration. Meta-analyses suggest that longer programs (eg, 4 weeks or longer) yield stronger and more consistent effects on anxiety and cognitive symptoms [51]. In our case, the 1-week period may have been insufficient to shift deeper cognitive patterns, such as maladaptive metacognitive beliefs. Alternatively, the rather moderate baseline levels of stress of our sample may have reduced the effectiveness of the intervention. A marginal trend toward reduced negative metacognitive beliefs in the DM group provides a promising direction for future studies using longer timelines or higher-intensity delivery formats.

Additionally, the qualitative data showed that while participants often described the exercises as calming, many also reported difficulties with concentration, repetitiveness, or a mismatch between the intervention and their immediate emotional needs. For instance, some participants in the DM group reported that attempting to observe stress-related thoughts without reacting could initially increase distress, particularly when the technique was not fully understood or when stressors felt highly salient. These experiences suggest that late adolescents may require more structure, guidance, or scaffolding to benefit optimally from cognitive or metacognitive techniques, especially in short interventions.

In contrast to the DM, the SB intervention did not lead to significant changes in stress, anxiety, or metacognitive beliefs compared with the passive control group. Although previous studies have shown that slow diaphragmatic breathing enhances parasympathetic regulation and reduces psychological stress, these benefits are typically observed in studies using longer or more structured training programs [26,52-54]. This suggests that the brief, 1-week practice period in our study may have been insufficient for late adolescents to experience meaningful psychological improvements. Moreover, similar to the DM intervention, the lack of significant findings may reflect the relatively low baseline stress and anxiety levels in our sample, limiting room for improvement (ie, floor effects).

Adherence and engagement emerged as critical factors across both data sources. Quantitatively, greater effort and more favorable perceptions of usability, technology acceptance, and outcome expectancy were associated with better outcomes, particularly in the SB group. Late adolescents who found the intervention easier to use and who expected it to be helpful reported greater reductions in anxiety. In the DM group, higher technology acceptance predicted greater stress reduction, suggesting that perceiving the intervention as relevant or useful may be especially important for benefiting from metacognitive strategies.

These patterns were strongly echoed in the qualitative data. Participants frequently described forgetting to complete the exercises, doing them late at night as an afterthought, or rushing through them without full attention. Several participants explicitly noted that they benefited more on days when they were able to focus and less when distracted or unmotivated. This convergence highlights that engagement and effort were associated with better outcomes, potentially outweighing previous familiarity with digital interventions, which did not predict outcomes quantitatively. Rather than general digital experience, participants’ motivation, expectations, and perceived relevance of the specific intervention were associated with better outcomes.

Taken together, the integrated findings suggest that brief digital interventions show preliminary promise for reducing perceived stress in late adolescents, particularly when they are experienced as engaging, credible, and personally relevant. However, achieving broader changes in anxiety and metacognitive beliefs may require longer intervention periods, greater personalization, and enhanced support for motivation and adherence. Future implementations may benefit from adaptive features that tailor content to the user’s emotional state, provide timely reminders, offer brief check-ins to support, and create a sense of togetherness for sustained engagement. In sum, while the interventions showed promising stress-related effects, the mixed methods evidence underscores that real-world effectiveness in adolescent populations depends as much on engagement and context as on intervention content itself.

### Exploratory Daily Intervention Use

While overall symptom trajectories across the 1-week period were modest and sometimes flat, our daily analyses revealed that participant engagement, specifically the self-reported effort invested in each session, played a meaningful role in intervention effectiveness. In both, the SB and DM groups, greater total effort was associated with improvements in specific outcomes. For SB, higher effort was linked to greater reductions in daily stress and negative metacognitive beliefs, suggesting that consistent engagement with this relaxation-based intervention can yield emotional benefits, even when average group-level change is limited. Similarly, for DM, effort was associated with lower daily anxiety, indicating that participants who applied themselves more fully to the metacognitive exercise experienced stronger short-term gains. These findings underscore the importance of designing interventions that not only appeal to youth but also sustain motivation and involvement over time.

### Limitations

Several limitations must be acknowledged. First, the 7-day intervention period was relatively short, which may have been insufficient for SB to produce measurable psychological effects. In addition, the absence of follow-up assessments leaves it unclear whether the benefits of DM would persist over time. Second, the passive control condition did not include any matched activity or engagement, making it impossible to rule out nonspecific effects such as expectation, structured daily routine, or simply taking a brief pause. Third, attrition was substantial, which may limit the generalizability of our findings and reflects the ongoing challenge of maintaining engagement in digital interventions. Also, our participants all being university students with 92% (n=104) female limits the generalizability of the results. Fourth, while focus groups provided valuable qualitative insight, participants may have been influenced by group dynamics, potentially limiting the generalizability of their responses. Fifth, baseline stress levels differed in the DM group compared with the SB and control group. Although the sensitivity analysis confirmed that the stress reduction effect remained significant after controlling for baseline stress, the small effect size and regression to the mean cannot be entirely excluded as a contributing factor. Sixth, all outcomes relied on self-report, and daily measures used 3-point scales which may have lacked sensitivity to detect nuanced change. Seventh, participants who completed fewer than 6 intervention days were excluded from the analyses. If these noncompleters differed systematically from completers, this may have introduced selection bias toward a more motivated or engaged sample. Finally, many of the participants experienced a relative delay between completing the intervention and filling out the postintervention questionnaire, which could potentially have influenced results, as circumstances could have changed between these periods.

Future studies should build on these findings by testing enhanced and adaptive DMHI designs in larger, more diverse samples and samples with elevated stress levels. Additionally, future studies should include an active control condition matched for time, digital format, and level of participant contact to allow for stronger causal inference regarding intervention-specific effects. Integrating personalization features and extending the intervention period could help sustain engagement and maximize effectiveness. Moreover, combining self-report with objective measures of stress and intervention use would strengthen the evidence base. Finally, given the documented rise in mental health concerns among adolescents [55,56], if replicated in larger and more diverse samples, DMHIs may offer a scalable approach to promoting youth well-being in educational settings.

### Conclusion

In conclusion, this study provides early formative evidence supporting the feasibility and usability of brief digital SB and DM interventions in a nonclinical sample of late adolescents from a single center. A preliminary signal for short-term stress reduction was observed for DM, although the absence of effects on anxiety and metacognitive beliefs, combined with the short 1-week intervention duration, predominantly female sample, and lack of follow-up, underscores the need for replication in larger, more diverse samples before any conclusions about effectiveness can be drawn. Beyond outcome effects, qualitative findings suggest that late adolescents value accessibility, autonomy, and noncompetitive features, offering concrete guidance for future intervention development. Together, these findings underscore the need to move beyond designing solely effective exercises to also designing for motivation. Strategies, such as gamification, social accountability, adaptive feedback, or ecological momentary prompts, may enhance engagement and foster more consistent use. Although effects were modest and the study faced limitations such as a relatively short intervention period and an incomplete sample, the integration of quantitative and qualitative evidence provides a nuanced understanding of both mechanisms of change and user experience in a youth sample. By prioritizing usability and motivation, and by aligning intervention design with late adolescents’ unique preferences and needs, DMHIs hold substantial promise as scalable solutions for addressing the growing mental health challenges facing young people today.

## Supplementary material

10.2196/94578 Multimedia Appendix 1 Exploratory Bayesian analyses of the daily intervention effects.

10.2196/94578 Multimedia Appendix 2 The focus group manual.

10.2196/94578 Multimedia Appendix 3 Analyses controlling for SUS, TAM, and outcome expectancy.

10.2196/94578 Multimedia Appendix 4 Descriptive statistics table of all outcome variables.

## Abbreviations

DM: detached mindfulness
DMHI: Digital mental health intervention
HRV: heart rate variability
SB: slow breathing
SUS: System Usability Scale
TAM: technology acceptance model
