# Supplementary material for: Effectiveness and Usability of Digital Slow Breathing and Detached Mindfulness Interventions in Late Adolescents: Mixed Methods Study
Source: JMIR Form Res. 2026 Sep 22;10:e94578. doi: 10.2196/94578 (PMC13597022; doi:10.2196/94578)
Supplement: Supplementary file 1 [file formative-v10-e94578-s002.pdf]

**Gesprekstechnieken**	**GEEN/NIET**<br>• oordelen, aannames en adviezen (laat OMA thuis)<br>• te veel reageren met 'heel goed', 'fantastisch' ect.<br>• technische termen gebruiken<br>• te schools gaan doen.<br>• te veel verschillende visies samen te vatten, doe dit alleen wanneer je het idee hebt dat je iemand niet helemaal goed begrijpt (begrijp ik het goed dat….).<br>**WEL**<br>**Interactie bevorderen**: verschillende mensen uitnodigen om te reageren, andere meningen vragen. *Herkennen jullie dat? Hoe is dat voor de anderen*? Maak oogcontact en benoem wanneer mogelijk namen.<br>**Vragen stellen:** Stel open vragen, wat en hoe vragen (pas op met waarom vragen).<br>**Doorvragen:** vertel eens? Hoe ging dat? Wil je dat toelichten?<br>**Stiltes:** stiltes mogen vallen (plan soms een 3 seconde stilte in)<br>**Loslaten**: houd niet te rigide vast aan je draaiboek. Afdwalen/andere volgorde is niet erg, als je de groep maar weer terugkrijgt op het onderwerp<br>**Houding:** reflecteer op je eigen non-verbale communicatie. Zorg voor een open en ontspannen houding.<br>**Notities:** schrijf eventueel tijdens de focusgroep belangrijke punten op waar je later op terug wil komen.
**Begin**	Goede middag allemaal! Allereerst bedankt voor het nemen van de tijd om deel te nemen aan dit onderzoek. Mijn naam is [NAAM] en ik ben hier namens het onderzoeksteam van de universiteit leiden om deze groep met jullie te leiden. Om mij te assisteren is hier ook [NAAM] ook namens ons onderzoeksteam. Jullie zijn uitgenodigd voor dit laatste deel van het onderzoek om jullie ervaringen met de oefening te delen, zodat wij de oefening kunnen verbeteren. Het gesprek zal maximaal 1.5 uur duren. Er ligt eten en drinken op tafel dus voel je vrij om dat gedurende het gesprek te pakken.<br>Ik zal jullie nog wat meer vertellen over het gesprek:<br>• Er bestaat niet zoiets als een fout antwoord, het is geen test, we zijn gewoon geïnteresseerd in jullie mening<br>• Iedereen mag zijn eigen mening hebben. Voel je daarom ook vrij om deze mening te geven ook als die anders is dan de rest. We zouden jullie willen vragen om elkaars antwoorden dan ook te respecteren.<br>• Ik wil jullie vandaag ook vooral uitnodigen om met ELKAAR in gesprek te gaan. Dus, als je op iemand wilt doorvragen of wilt reageren voel je vrij om dat te doen. Je hoeft dus niet alleen op mij te reageren. Ik ben hier om vragen te stellen, te luisteren en ervoor zorgen dat iedereen zijn mening kan delen. Wij zijn geïnteresseerd in de ervaringen van jullie allemaal.<br>• Wij nemen het gesprek op met een audiorecorder (wijs aan) omdat we niks van jullie waardevolle commentaren willen missen. We gaan deze opnames volledig uitschrijven en zullen daarbij jullie namen volledig verwijderen.<br>**Hebben jullie nog vragen voordat we beginnen?**

Introductie	Jullie zaten allemaal in de rustig ademen conditie. Wij willen graag samen met jullie hulp de interventie verbeteren. Jullie hebben zelf 7 dagen kennisgemaakt en geoefend met rustig ademen. In deze groepsbijeenkomst zullen wij bespreken hoe jullie de interventie hebben ervaren. Als je het handig vindt kun je je notities erbij pakken.<br>OF<br>Jullie zaten allemaal in de mindfulness conditie. Wij willen graag samen met jullie hulp de interventie verbeteren. Jullie hebben zelf 7 dagen kennisgemaakt en geoefend met mindfulness. In deze groepsbijeenkomst zullen wij bespreken hoe jullie de interventie hebben ervaren. Als je het handig vindt kun je je notities erbij pakken.	Oké laten we beginnen met een eerste ronde waarbij je je naam noemt en de volgende zin afmaakt: Toen ik de interventie voor het eerst gebruikte merkte ik dat ik……………<br><br>Oké laten we beginnen met een eerste ronde waarbij je je naam noemt en de volgende zin afmaakt: Toen ik de interventie voor het eerst gebruikte merkte ik dat ik……………
Effectiviteit	Ik hoor dat jullie ervaringen heel wisselend zijn (of juist heel erg vergelijkbaar). [Deelnemer x], mag ik eens bij jou beginnen? Jij zei dat ………………………….. Kun je dat toelichten?	Hoe is dat voor de anderen?<br>Welk gedeelte van de oefening hebben jullie als behulpzaam ervaren?<br>Welk gedeelte van de oefening hebben jullie als frustrerend ervaren?
Gebruiksvriendelijkheid	Voor ons is het heel belangrijk dat de interventie gebruiksvriendelijk is. Bijvoorbeeld dat het makkelijk is om de oefeningen toe te passen. Hoe was dat voor jullie? Hoe gemakkelijk of moeilijk was het voor jullie om de oefening een week lang uit te voeren?	Waardoor ervaarden jullie moeite/gemak om de oefening elke dag uit te voeren?<br><br>[als van toepassing] Waardoor verloor je interesse in de oefening?
	[Indien niet genoemd:] En bij gebruiksvriendelijkheid hoort eigenlijk ook nog hoe duidelijk alles was. Was het over het algemeen duidelijk wat jullie moesten doen?	Zo nee, wat was er niet duidelijk?<br>Waarom niet?<br>Wat kunnen we daaraan verbeteren?
Vormgeving	Wat vonden jullie van de vormgeving?	Wat vonden jullie aantrekkelijk aan de vormgeving van de interventie?<br>Wat vonden jullie minder aantrekkelijk aan de vormgeving van de interventie?
OPTIONEEL ALS ER MENSEN ZIJN DIE DE INTERVENTIE NIET HEBBEN GEVOLGD	Bij een minderheid mag alleen de eerste vraag gesteld worden. Bij een meerderheid mag je wel alle vragen onder OPTIE B stellen.	1: Wat is de reden dat je de interventie niet hebt gevolgd?’
Motivatie niet uitvoeren oefening	Wat is de reden dat je de interventie niet hebt gevolgd?	Wat maakt dat je geen zin/ het te druk etc. ervoor had?

	Wat had wij anders kunnen doen, waardoor het makkelijker was om het uit te voeren?<br><br>Welke veranderingen hoop je te zien in jouw leven of bij jongeren in het algemeen als gevolg van deze interventie?	Wat zou mooi zijn om te bereiken met de interventie?
Perceptie vraag	Kun je me vertellen wat je denkt dat deze interventie inhoudt, gebaseerd op wat je tot nu toe hebt gehoord?	Wat spreekt je daarbij aan of wat spreekt je daarbij niet aan?
Apps mentale gezondheidsoefeningen	Wat vinden jullie van het idee van apps met mentale gezondheidsoefeningen?	Hebben jullie wel eens een app gebruikt met mentale gezondheidsoefeningen? War vond je daar wel of niet prettig aan?<br><br>Vinden jullie een app fijner dan een oefening met een behandelaar erbij?
Advies voor app	Echt heel fijn dat jullie al zoveel informatie met mij en elkaar hebben gedeeld. Om dit gesprek af te sluiten zou ik graag nog even willen inventariseren wat voor ideeën jullie hebben over een bijbehorende app. Stel je voor dat je advies mag geven aan de makers van de interventie om een app om de oefening heen te bouwen voor jou ideale ervaring.	Wat voor eigenschappen of extra functies zou je dan in een bijbehorende app willen zien? Je kunt dan denken aan dingen om de oefening leuker te maken, of om het eenvoudiger te maken, maar alle suggesties zijn welkom.
	Nu zijn er nog wat mogelijke aanpassingen die jullie niet hebben genoemd die ik nog even bij jullie wil checken. Misschien kunnen jullie je hand opsteken als je dit graag zou willen:<br>[Vraag aan één iemand die zijn hand opstak bij wel en aan één iemand die zijn hand opstak bij niet of ze dat kunnen uitleggen.]	Wie van jullie zou het fijn vinden<br>• als je je eigen vooruitgang bij kan houden?<br>• als er een chat functie is om vragen te stellen over je mentale gezondheid en de Oefening (Chat AI)? In de vorm van een avatar?<br>• als er een puntensysteem bijzit om te vergelijken met andere gebruikers (Gamificatie)?<br>• om met anderen te interacteren die op dat moment ook de oefening uit voeren? (Peer system)"
Afsluiting	Hebben wij iets gemist wat nog niet genoemd is?<br><br>Bedankt voor jullie tijd en voor het beantwoorden van onze vragen! Als er iets is wat je nog graag had willen delen en waar we vandaag geen tijd meer voor hadden dan kan je dat altijd doen door nog te mailen naar het volgende email adres die staat op deze afsluitingsformulieren (deel nu het debriefing formulier uit).	
