# Supplementary material for: Effectiveness and Usability of Digital Slow Breathing and Detached Mindfulness Interventions in Late Adolescents: Mixed Methods Study
Source: JMIR Form Res. 2026 Sep 22;10:e94578. doi: 10.2196/94578 (PMC13597022; doi:10.2196/94578)
Supplement: Supplementary file 2 [file formative-v10-e94578-s001.docx]

**Multimedia Appendix 2**

**Methods**

**Measures**

***Effort***

To measure effort, the Dutch Inzettool was used as a daily measure after doing the intervention [1]. This tool was filled out after each intervention day and is a short 6-item questionnaire about their effort in performing the intervention exercises. We took three items from this questionnaire related to our brief intervention: “Today I liked doing the exercise”, “Today it was easy to stay focused on the exercise”, and “Today I did my best”. The three items were calculated to a total score. The three questions were scored on a 3-point Likert scale, ranging from not at all to very much. A higher score indicates a higher effort.

***Stress, anxiety, and negative metacognitive thoughts***

Students filled out four additional daily items. First, before participants received the intervention, they were asked whether the day was stressful. After the intervention, participants are asked to what extent their stress, anxiety, and negative thoughts had decreased. The four questions were scored on a 3-point Likert scale, ranging from not at all to very.

**Data analyses**

To evaluate change in participants’ perceived stress, anxiety, and negative metacognitive beliefs across the 7-day intervention period, we employed Bayesian multilevel models. This approach was chosen over the initially planned traditional frequentist mixed-effects models due to its increased robustness in small to moderate samples, its ability to flexibly capture individual trajectories, and its direct estimation of uncertainty through credible intervals. Each outcome was modeled as a function of time, using both linear and quadratic terms (i.e., day and day²) to capture potential non-linear patterns in change (e.g., rapid early improvement followed by a plateau). Random intercepts and slopes were estimated for each participant to account for between-person differences in baseline levels and trajectories of change. In addition to time, we examined the role of Total Effort as a continuous predictor. Importantly, we tested whether the effect of day on outcomes was moderated by effort, by including an interaction term between day and Total Effort. This allowed us to test whether the rate of improvement over the week was greater for participants who reported higher levels of engagement with the intervention. All models were estimated using the brms package in R, which interfaces with Stan to conduct full Bayesian inference via Markov Chain Monte Carlo (MCMC) sampling. Each model was run with four chains, 4,000 iterations per chain (with 1,000 warm-up iterations), yielding 12,000 post-warmup samples. Default weakly informative priors were used. Convergence was assessed using the R-hat statistic (values < 1.01), effective sample sizes (ESS), and inspection of trace plots. Model estimates are reported as posterior means with 95% credible intervals (CI).

**Results**

**Bayesian Daily Intervention Effects: Slow breathing**

To assess the effectiveness of the slow breathing intervention over one week, we fitted Bayesian multilevel models predicting daily levels of stress, anxiety, and negative metacognitive beliefs, including linear and quadratic effects of time and the main effect of total effort.

For stress, there was moderate evidence for a small quadratic improvement across the week (see Figure MA1). The linear effect of time was uncertain (Estimate = 0.09, 95% CI [−0.35, 0.53]), while the quadratic effect trended negative (Estimate = −0.02, 95% CI [−0.05, 0.01]), suggesting a possible plateau in stress reduction toward the end of the intervention period. Although the credible interval for the quadratic effect slightly overlapped zero, the pattern indicates that early gains may have leveled off. Higher total effort was associated with greater reductions in stress, suggesting that participants who consistently engaged with the intervention tended to experience stronger benefits.

For anxiety and negative metacognitive beliefs no evidence was found for a systematic change, neither linear (b_anxiety_ = –0.04, 95% CI [–0.48, 0.40], and b_negative metacognitive beliefs_ = 0.15, 95% CI [–0.32, 0.61]) or quadratic (b_anxiety_ = –0.01, 95% CI [–0.04, 0.02], and b_negative metacognitive beliefs_ = –0.01, 95% CI [–0.05, 0.02]), across the week. These findings suggest that the slow breathing intervention did not produce consistent short-term improvements in anxiety during the study period. However, higher total effort was credibly associated with lower levels of daily negative metacognitive beliefs (b = 0.11, 95% CI [0.01, 0.22]). Participants who invested more effort into the intervention tended to experience modest but consistent reductions in metacognitive negative metacognitive beliefs, independent of the day of intervention. In summary, the slow breathing intervention appeared beneficial for reducing stress and negative metacognitive beliefs, particularly among participants who reported putting in more effort. There was no clear evidence of improvement in anxiety over the 7-day period.

**Figure MA1**
*Mean Daily Stress Improvement Scores across the 7-day intervention period in the Slow Breathing Group. Error Bars Represent 95% confidence intervals (N = 38)*

*Note.* While slight fluctuations were observed, the overall trend suggests a modest, curvilinear pattern, with anxiety improvement peaking around Day 4 and tapering off toward the end of the week. Variability increased across days, indicating individual differences in response patterns.

**Bayesian Daily Intervention Effects: Detached Mindfulness**

To examine daily changes in stress, anxiety, and negative metacognitive beliefs during the detached mindfulness intervention, Bayesian mixed-effects models were estimated including linear and quadratic terms for day, total effort, and their interaction, with random slopes and intercepts.

Across all outcomes, the linear effects of day were small and uncertain: for stress (Estimate = 0.01, 95% CI [−0.41, 0.43]), anxiety (Estimate = 0.26, 95% CI [−0.13, 0.65]), and negative metacognitive beliefs (Estimate = −0.07, 95% CI [−0.49, 0.36]). The quadratic term trended negative in all models, indicating a possible curvilinear pattern, with strongest support for anxiety (Estimate = −0.04, 95% CI [−0.07, −0.01]). Total effort was positively associated with anxiety reduction (Estimate = 0.12, 95% CI [0.04, 0.21]), while the effects for stress (Estimate = 0.07, 95% CI [−0.03, 0.16]) and negative metacognitive beliefs (Estimate = 0.04, 95% CI [−0.05, 0.13]) showed greater uncertainty. These findings suggest that while the detached mindfulness intervention may offer some benefit for anxiety (see Figure MA2), particularly when participants are actively engaged, the overall pattern of change was modest, and less consistent for other outcomes.

**Figure MA2**
Mean daily anxiety improvement scores across the 7-day intervention period in the detached mindfulness group. Error bars represent 95% confidence intervals.

*Note.* While slight fluctuations were observed, the overall trend suggests a modest, curvilinear pattern, with anxiety improvement peaking around Day 4 and tapering off toward the end of the week. Variability increased across days, indicating individual differences in response patterns.

**References**

1. Voortgezet leren (n.d.). *Inzettool*. Retrieved November 23, 2023 from [Inzettool - Voortgezet Leren](https://www.voortgezetleren.nl/post/inzettool/)
