# Supplementary material for: Effectiveness and Usability of Digital Slow Breathing and Detached Mindfulness Interventions in Late Adolescents: Mixed Methods Study
Source: JMIR Form Res. 2026 Sep 22;10:e94578. doi: 10.2196/94578 (PMC13597022; doi:10.2196/94578)
Supplement: Supplementary file 3 [file formative-v10-e94578-s003.docx]

**Multimedia Appendix 3**

**Methods**

**Measures**

***System Usability***

System usability was measured during post-test with the Dutch and adapted version of the System Usability Scale (SUS)[1]. The questions (e.g., “I would use this intervention often if I had access to it.”) were adapted so that they are not related to systems but to our intervention videos. Participants had to indicate on 9 items (e.g., to what extent they thought the intervention videos were usable, scored by a 5-point Likert scale from 1 "strongly disagree" to 5 "strongly agree" (α = .72). A higher score indicates a higher system usability.

***Technology Acceptance***

Technology acceptance was measured during post-test with 5 items related to this subscale in the Technology Acceptance Model (TAM)[2]. The questions (e.g., “I think that using this intervention would decrease my stress levels”) were adapted to relate to the intervention videos [3]. The items were formulated as statements scored by a 5-point Likert scale from 1 "strongly disagree" to 5 "strongly agree" (α = .87). A higher score indicates a higher technology acceptance.

***Outcome expectancy***

Based on the model of Rodgers & Brawley (1991), a 1-item questionnaire was administered pre-test, according to the modified measure of Cramp & Brawley (2009). The item for this measures the expected helpfulness of the intervention outcome. The likelihood of this outcome is measured on a 7-point Likert scale (-3=*not helpful at all* to 3=*very helpful*).

**Data analysis**

To explore whether perceived usability, technology acceptance, internet usage, or familiarity with digital interventions predicted intervention effectiveness, we conducted multiple linear regressions on change scores (post - pre) for each dependent variable within each intervention group separately, with age entered as a covariate. We initially planned to include SUS and TAM scores as covariates in the main mixed-effects models. However, because these measures were only collected for the two intervention groups, including them would have excluded all control participants and reduced power. As SUS and TAM are post-intervention ratings, we instead analyzed them in separate regressions within the intervention groups, using pre–post change scores as outcomes. This allowed us to retain the full sample in the main analyses while exploring their role as predictors of individual differences in change.

**Results**

**Controlling for SUS, TAM, and outcome expectancy**

To examine whether individual differences in perceived usability and technology acceptance predicted intervention effectiveness, we conducted six separate linear regression analyses using change scores (post – pre) as dependent variables. Three analyses were restricted to participants in the slow breathing group (*N* = 38), and the other three for the detached mindfulness group (*N* = 38) and were selected due to the small sample size per condition. We modeled change scores for stress, anxiety, and negative metacognitive beliefs separately, each time including either system usability or technology acceptance (centered), along with age as a covariate.

Within the slow breathing group, linear regression analyses were conducted to examine whether perceived usability, technology acceptance and outcome expectancy predicted individual differences in pre–post change scores for stress, anxiety, and negative metacognitive beliefs. Usability scores were significantly associated with greater reductions in anxiety (β = 0.29, *p* = .021), explaining approximately 14% of the variance. In contrast, SUS scores were not significantly related to changes in stress (β = 0.71, *p* = .262) or negative metacognitive beliefs (β = 0.08, *p* = .394), with both models explaining minimal variance. No significant associations were observed between technology acceptance scores and greater reductions in stress, anxiety, and negative metacognitive beliefs (all *p* values > .108)

We examined whether participants’ outcome expectancy predicted pre–post changes in stress, anxiety, and negative metacognitive beliefs within the slow breathing group. Outcome expectancy was significantly associated with anxiety reduction (β = 0.71, *p* = .028), accounting for approximately 13% of the variance in change scores. This suggests that participants who expected greater benefits from the intervention tended to show stronger decreases in anxiety. However, outcome expectancy was not significantly related to changes in stress (β = 1.29, *p* = .429) or negative metacognitive beliefs (β = 0.22, *p* = .350), with both models explaining little to no variance.

For the detached mindfulness group, we found that perceived usability and outcome expectancy scores were not significantly associated with any of the outcomes. However, technology acceptance scores significantly predicted reductions in stress (β = 1.64, *p* = .033), indicating that participants who perceived the intervention as more useful or acceptable reported greater reductions in stress from pre- to post-intervention. No significant associations were found between TAM and changes in anxiety or negative metacognitive beliefs.

**Controlling for internet usage and familiarity with seeking digital interventions**

Lastly, we explored the role of internet usage and familiarity seeking digital interventions as possible moderators for the intervention effects. We ran the same analyses similarly as for the perceived usability and technology acceptance and found that internet usage and familiarity with digital interventions were not significant predictors in any of the models (all *p* > .699)

**References**

1. Brooke J. SUS: a quick and dirty usability scale. In: *Usability Evaluation in Industry*. London: Taylor & Francis; 1996:189-194.
2. Davis FD. Technology acceptance model: TAM. In: Al-Suqri MN, Al-Aufi AS, eds. *Information Seeking Behavior and Technology Adoption*. Hershey, PA: IGI Global; 1989:205-219.
3. Saadé R, Bahli B. The impact of cognitive absorption on perceived usefulness and perceived ease of use in online learning: an extension of the technology acceptance model. *Inf Manag*. 2005;42(2):317-327.
