# Supplementary material for: Effectiveness and Usability of Digital Slow Breathing and Detached Mindfulness Interventions in Late Adolescents: Mixed Methods Study
Source: JMIR Form Res. 2026 Sep 22;10:e94578. doi: 10.2196/94578 (PMC13597022; doi:10.2196/94578)
Supplement: Supplementary file 4 [file formative-v10-e94578-s004.docx]

**Multimedia Appendix 1**

**Table MA1**

*Descriptives Statistics Table of All Outcome Variables*

|  |  | **Stress** | | **Anxiety** | | **Negative metacognitive beliefs** | | **System Usability** | | **Technology Acceptance** | | | **Familiarity mental health tools** | | **Internet usage** | |  |
| --- | --- | --- | --- | --- | --- | --- | --- | --- | --- | --- | --- | --- | --- | --- | --- | --- | --- |
| **Overall model** | **Phases** | Mean | SD | Mean | SD | Mean | SD | Mean | SD | | Mean | SD | Mean | SD | Mean | SD |  |
|  | Pre-intervention | 58.20 | 19.43 | 22.63 | 4.86 | 14.18 | 4.68 | - | - | | - | - | 34.40 | 5.59 | 14.98 | 2.05 |  |
|  | Post-intervention | 52.76 | 22.05 | 21.66 | 4.68 | 13.82 | 4.38 | - | - | | - | - | - | - | - | - |  |
| **Control** |  | | | | | | | | | | | |  |  |  | |  |
|  | Pre-intervention | 52.324 | 17.792 | 22.351 | 5.240 | 13.65 | 4.95 | - | - | | - | - | 35.67 | 5.39 | 14.74 | 1.82 |  |
|  | Post-intervention | 50.167 | 26.614 | 21.472 | 5.180 | 13.36 | 5.07 | - | - | | - | - | - | - | - | - |  |
| **Detached mindfulness** |  |  |  |  |  |  |  |  |  | |  |  |  |  |  |  |  |
|  | Pre-intervention | 61.37 | 20.97 | 22.05 | 4.84 | 14.08 | 4.43 | - | - | | - | - | 32.26 | 6.63 | 15.15 | 2.14 |  |
|  | Post-intervention | 55.84 | 21.08 | 21.24 | 4.48 | 14.18 | 4.11 | 36.6 | 4.14 | | 15.9 | 4.37 | - | - | - | - |  |
| **Slow breathing** |  |  |  |  |  |  |  |  |  | |  |  |  |  |  |  |  |
|  | Pre-intervention | 61.78 | 18.03 | 23.82 | 4.30 | 15.04 | 4.72 | - | - | | - | - | 34.63 | 4.59 | 15.11 | 2.24 |  |
|  | Post-intervention | 51.89 | 16.15 | 22.52 | 4.69 | 13.93 | 3.81 | 36.3 | 4.70 | | 17.3 | 4.59 | - | - | - | - |  |

*Note.* The table shows the main outcome variables' total scores and standard deviations per timepoint. First for the overall model and later per condition. All data was summarized over 114 participants in total.
